# Modelling cassava production and pest management under biotic and abiotic constraints

**DOI:** 10.1007/s11103-021-01170-8

**Published:** 2021-07-27

**Authors:** Vasthi Alonso Chavez, Alice E. Milne, Frank van den Bosch, Justin Pita, C. Finn McQuaid

**Affiliations:** 1 Department of Biointeractions and Crop Protection, Rothamsted Research, Harpenden, AL5 2JQ UK; 2 School of Molecular and Life Sciences, Curtin University, GPO Box U1987, Perth, WA 6845 Australia; 3 Laboratory of Plant Physiology, Université Félix Houphouët-Boigny, Abidjan, Côte d’Ivoire; 4 Department of Infectious Disease Epidemiology, London School of Hygiene & Tropical Medicine, London, UK

**Keywords:** Modelling, Cassava, Pests, Dynamics, Surveillance, System

## Abstract

**Key message:**

We summarise modelling studies of the most economically important cassava diseases and arthropods, highlighting research gaps where modelling can contribute to the better management of these in the areas of surveillance, control, and host-pest dynamics understanding the effects of climate change and future challenges in modelling.

**Abstract:**

For over 30 years, experimental and theoretical studies have sought to better understand the epidemiology of cassava diseases and arthropods that affect production and lead to considerable yield loss, to detect and control them more effectively. In this review, we consider the contribution of modelling studies to that understanding. We summarise studies of the most economically important cassava pests, including cassava mosaic disease, cassava brown streak disease, the cassava mealybug, and the cassava green mite. We focus on conceptual models of system dynamics rather than statistical methods. Through our analysis we identified areas where modelling has contributed and areas where modelling can improve and further contribute. Firstly, we identify research challenges in the modelling developed for the surveillance, detection and control of cassava pests, and propose approaches to overcome these. We then look at the contributions that modelling has accomplished in the understanding of the interaction and dynamics of cassava and its’ pests, highlighting success stories and areas where improvement is needed. Thirdly, we look at the possibility that novel modelling applications can achieve to provide insights into the impacts and uncertainties of climate change. Finally, we identify research gaps, challenges, and opportunities where modelling can develop and contribute for the management of cassava pests, highlighting the recent advances in understanding molecular mechanisms of plant defence.

## Introduction

Cassava, *Manihot esculenta* (Euphorbiaceae) is a vegetatively propagated tuber crop originating in Brazil that was introduced to Africa in the sixteenth century and Asia in the eighteenth century (Thottappilly et al. 2006). Today, cassava is grown in more than 39 African and 56 other countries around the world (Thottappilly et al. 2006) and has become the staple food crop of approximately 800 million people worldwide (Tomlinson et al. 2018). Some of the reasons for the widespread cultivation include that it can be grown throughout the year, it is highly tolerant to drought and it can grow even in poor soil conditions (Tomlinson et al. 2018). Additionally, while other crops are projected to be negatively impacted by climate change in Africa, cassava is expected to be positively impacted (Jarvis et al. 2012).

Besides being a staple for food consumption, cassava is also used for the manufacturing of pharmaceutical products, as livestock feed and as biofuel (Alene et al. 2018).

Pests and diseases pose a serious threat to cassava, whether endemic or introduced. Endemic syndromes and diseases include the prominent cassava frogskin disease (CFSD) syndrome in Latin America. Although this disease was first identified in the 1970s, identifying the causal agent has been challenging (Calvert et al. 2012; Legg et al. 2015). Recent evidence shows that the disease is associated with several viruses and phytoplasmas (Calvert et al. 2012; Legg et al. 2015). Other relevant diseases are Cassava Mosaic Disease (CMD) caused by cassava mosaic geminiviruses (CMGs) in Africa and Asia, and Cassava Brown Streak Disease (CBSD) caused by cassava brown streak viruses (CBSVs) in Africa, which stand out as the main global threat to cassava production (Legg et al. 2014a). Introduced cassava arthropods and diseases date back to the 1970s when cassava mealybug (*Phenacoccus manihoti*) (CM), cassava green mite (*Mononychellus tanajoa*) (CGM), and cassava bacterial blight (CBB) caused by *Xanthomonas axonopodis* pv. *Manihotis* were introduced to Africa. Later on, CM and CBB were also introduced into southeast Asia (Legg et al. 2015). By 1970 CBB was found in most cassava growing areas in Central and South America, the Caribbean, Africa and Asia (Bradbury 1986).

To counteract the effects of arthropods, syndromes, and diseases (henceforth referred to collectively as pests) different control and prevention strategies can be followed. In this review we focus on various forms of modelling that have been used to simulate the effect of cassava pests to gain a greater understanding and optimise management.

We divide this review in 4 sections. Section 1 corresponds to the introduction which frames the structure and purpose of the paper. In Sect. Most relevant cassava pests and efforts to detect, control and eradicate them we summarise the most common cassava pests affecting agriculture worldwide and the efforts undertaken to detect, control and eradicate them. This provides the context necessary to develop Sect. Modelling approaches for the surveillance, detection and control of cassava pests where we focus on the modelling developed for the study of cassava pests’ surveillance, dynamics, and management.

In Sect. Modelling approaches for the surveillance, detection and control of cassava pests, we look at four different ways modelling has contributed to the mitigation of cassava pests’ effects. Firstly, we look at models developed to simulate the surveillance and detection of different pests in different geographies and scenarios, as we know that when a pest threatens a certain region where it has not yet been found, surveillance efforts should be prioritised to improve preparedness.

Once a pest is found for in a region, control programmes and mechanisms to eradicate and manage its spread are often implemented. How that implementation takes place, can be informed through modelling. Thus, we look at models that have provided insight about management strategies for different pests and in different conditions. We highlight success stories in the control and management of cassava pests and discuss reasons behind programmes failures.

Then, we summarise host-pest interaction models that help us better understand the mechanisms that can help the pest thrive and spread and the mechanisms that may limit their effect.

Finally, we look at the effects of climate change on the crop-pest system. Even when advances in the understanding of the system have been informing us for years, we know that the environmental conditions of these systems are changing. To overcome these challenges, we need to understand how these affect the crop system, the host-pest interactions, and their spread.

We finish the review highlighting research opportunities and challenges that modelling can take advantage of to counter the effect of cassava pests in Sect. Future challenges and opportunities.

## Most relevant cassava pests and efforts to detect, control and eradicate them

Cassava is affected by a variety of pests including viruses, bacteria, phytoplasmas, arthropods, nematodes and fungi. The greatest diversity of threats is found in Latin America; however, due to the endemicity of cassava to this region and the co-evolution of the host-pest systems, the impact of these threats to cassava in Latin America is generally smaller than in Africa and Asia. For a comprehensive list of cassava pests the reader can refer to Howeler et al. (2012); Graziosi et al. (2016); Rapisarda and Cocuzza (2017); McCallum et al. (2017). Table 1 summarises the most cited cassava pests and their acronyms used in this review.

**Table 1:** Names of cassava pests, their acronyms, and causal agents

Pest	Acronym	Causal agents	References
Cassava frogskin disease	CFSD	Phytoplasmas and cassava frogskin-associated viruses	(Calvert and Thresh 2002; Calvert et al. 2012; Legg et al. 2015)
Cassava common mosaic disease	CCMD	Cassava common mosaic virus (CsCMV)	(Calvert and Thresh 2002)
Cassava vein mosaic disease	CVMD	Cassava vein mosaic virus (CsVMV)	(Calvert and Thresh 2002)
Cassava mosaic disease	CMD	Cassava mosaic geminiviruses (CMGs)	(Legg et al. 2015)
Cassava brown streak disease	CBSD	Cassava brown streak virus (CBSV) and Ugandan cassava brown streak virus (UCBSV)	(Legg et al. 2015)
Cassava bacterial blight	CBB	*Xanthomonas axonopodis* pv. *manihotis* (*Xam*)	(Calvert and Thresh 2002; Graziosi et al. 2016)
Cassava mealybug	CM	*Phenacoccus manihoti* Mat.-Ferr	(Parsa et al. 2012; Graziosi et al. 2016)
Cassava green mite	CGM	*Mononychellus tanajoa* (Bondar)	(Parsa et al. 2015; Le et al. 2018)

### Virus diseases

Virus-caused diseases of economic importance in cassava in Latin America include cassava frogskin disease (CFSD), although as mentioned earlier, this disease may also involve phytoplasmas (Legg et al. 2015); cassava common mosaic disease (CCMD) caused by cassava common mosaic virus (CsCMV) and cassava vein mosaic disease (CVMD) caused by cassava vein mosaic virus (CVMV) (Calvert et al. 2012). Diseases caused by CsCMV and CVMV are usually of low importance but can cause significant losses when conditions are optimal. Nonetheless, rouging of infected plants appears to provide adequate control for both viruses. In addition, disinfection of harvesting tools helps to limit the spread of CsCMV (Calvert et al. 2012).

CFSD, meanwhile, can cause up to 90% yield losses, making it the most important cassava virus disease in Latin America (Calvert et al. 2012). The disease directly affects the roots, causing longitudinal fissures along the roots’ length. CFSD spreads mainly through infected cuttings or planting material, although the involvement of a vector may also be possible. Perhaps due to the difficulty in understanding the aetiology and virus species causing CFSD, to our knowledge, no modelling work on its’ dynamics, spread or control exists. Fortunately, several cassava varieties resistant to the disease exist, so through the use of disease-fee cuttings, phytosanitation measures and tolerant varieties the disease can be controlled (Calvert et al. 2012).

In Africa and Asia, the virus diseases with the greatest economic impact are CMD (in both continents) and CBSD (in Africa only). CMD is caused by a conglomerate of 9 geminiviruses (CMGs) and several variants vectored by the whitefly *Bemisia tabaci* in a persistent manner (Legg et al. 2015); of these, 7 are found in Africa and 2 in Asia. Besides it being vectored by *B. tabaci*, CMD and CBSD are spread by the planting of infected cuttings.

CMD causes mottling and yellow mosaic coloration on the leaves, leaf deformation and reduction in the size of leaves and plants (Alabi et al. 2011). In Africa, it has been calculated that the root yield losses range from 15 to 24% annually, equivalent to US$ 1–2.3 billion (Alabi et al. 2011; Szyniszewska et al. 2017). In Asia, CMD is relatively recent so little is known on the impacts to cassava productivity although average losses of 30% have been reported from India (Minato et al. 2019) and the incidence throughout South East Asia is rapidly increasing (CIAT 2019).

CBSD, on the other hand, is caused by two plant RNA-viruses occurring either together or separately (Legg et al. 2014b). The disease was initially confined to the East Coast of Africa but since 2004 it has rapidly spread westward (Legg et al. 2011; Tomlinson et al. 2018). The most economically important symptom of CBSD is the necrotic rot of the roots which can result in large yield losses. For example, across Kenya Tanzania, Uganda and Malawi moderately severe necrosis was found in 6–13% of the cassava roots examined. If yield losses are estimated at 8% of the 36 million tonnes produced in these regions the yield losses constitute about 3 million tons, valued at approximately US$750 million per year have been estimated (Hillocks and Maruthi 2015). The primary control strategies for CMD and CBSD have historically been breeding and deployment of resistant cassava varieties, phytosanitation such as roguing and selection of disease-free cuttings, cultural control approaches (e.g. timing of crop planting and intercropping), and vector control using insecticides or biocontrol (Legg et al. 2015; McCallum et al. 2017). Integrated management strategies can combine several of these tactics to make control more sustainable.

### Cassava bacterial blight

The causal agent of Cassava Bacterial Blight (CBB), the bacterium *Xanthomonas axonopodis* pv. *manihotis* (*Xam*) was discovered at the beginning of the century in South America and it was introduced into Africa in the 1970s (Boher and Verdier 1994). It is ranked as the 6th most serious bacterial pathogen in the world in the top 10 plant pathogenic bacteria in molecular plant pathology (Mansfield et al. 2012), as it can cause yield losses of 12–92% (Graziosi et al. 2016). The symptoms of CBB include water-like spots on the leaves and, at later stages of infection, wilting and defoliation (Graziosi et al. 2016; Fanou et al. 2018). Unfortunately, CBB’s causal agent has several means of survival and dissemination. These include survival on debris, on some weeds, and latently on cassava stems. Dispersal can also be aided by the grasshopper *Zonocerus variegatus* and human-mediated movement of infected stems (Fanou et al. 2018). Currently, no resistance genes have been demonstrated to be effective against CBB, and chemical methods are not an economically feasible form of control for smallholder farmers (Mutka et al. 2016). Some methods of control include intercropping, phytosanitation, clean seed systems and late planting dates (Fanou et al. 2018).

### Arthropod pests

At a global level, the most damaging arthropod pests of cassava are the cassava mealybug (*Phenacoccus manihoti*) (CM), and the cassava green mite (*Mononychellus tanajoa*) (CGM) although several other species of both mealybugs and mites have been reported to cause large yield losses in South East Asia (Graziosi et al. 2016).

The CM is endemic to the Paraguay river basin and was introduced to Africa in the 1980’s from the Americas (Neuenschwander et al. 1988) and it was first identified in Thailand in 2008. This pest has caused historical yield reductions of roughly 80% in some African regions, and up to 40% in Thailand (Graziosi et al. 2016; Wyckhuys et al. 2019a). In Africa the successful release of the host-specific parasitic wasp *Anagyrus lopezi* in 1981 permanently suppressed the mealybug (Wyckhuys et al. 2019a). In Asia, meanwhile, different control methods including the use of insecticides and biocontrol (including *A. lopezi*) are currently used (Aekthong and Rattanakul 2019), providing adequate control.

**Fig. 1:**
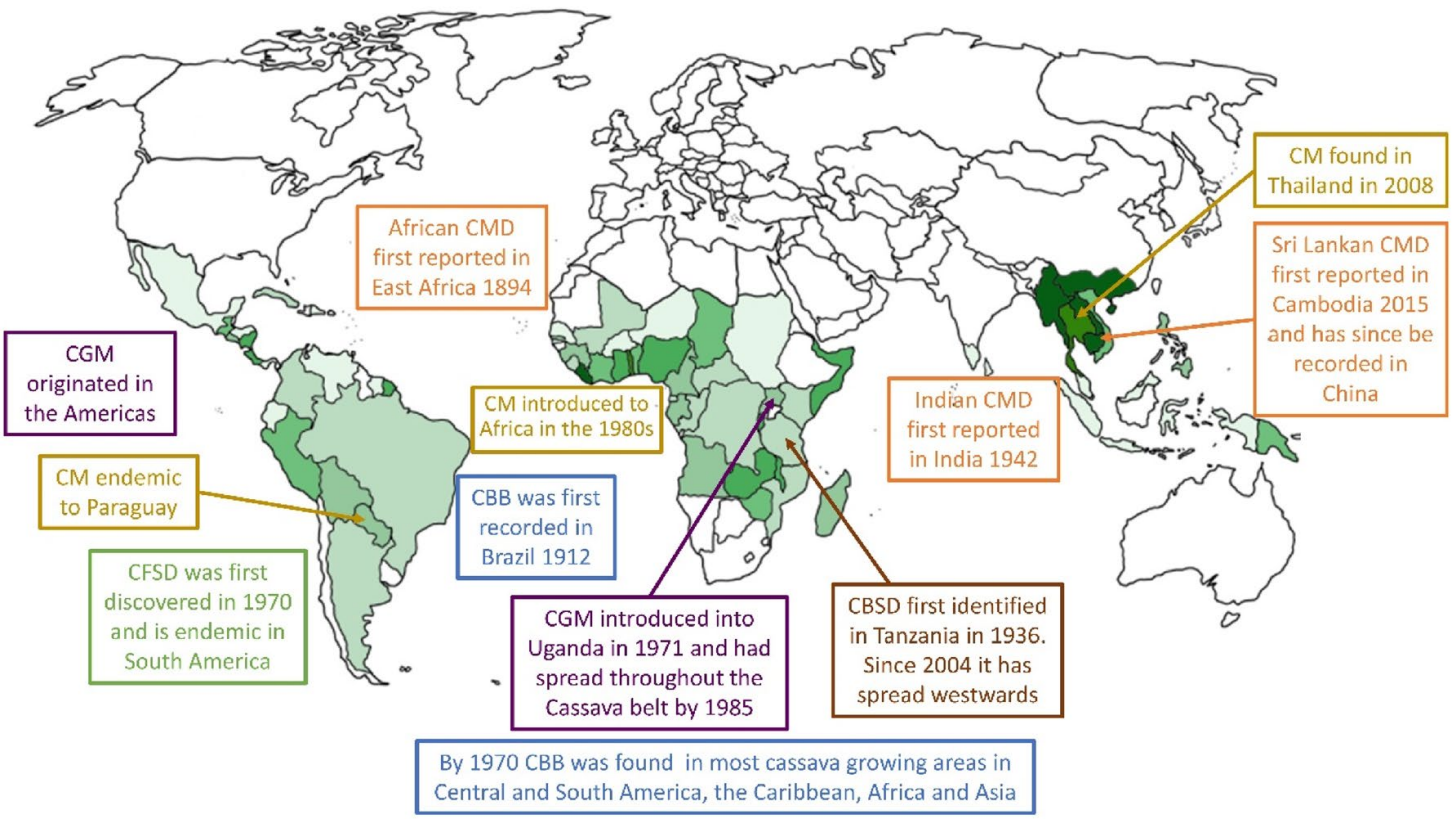
A map showing the cassava growing regions in the world (according to FAOSTAT, 2014) with indication of the geographical extent of the major cassava diseases: (i) Cassava Frogskin Disease (CFSD), (ii) Cassava Mosaic Disease (CMD), (iii) Cassava Brown Streak Disease (CBSD) (iv) Cassava Bacterial Blight (CBB), and arthropod-pests (v) Cassava Mealybug (CM) and (vi) the Cassava Green Mite (CGM)

The CGM, which also originated in the Americas, was firstly identified in Uganda in 1971 but it is now confirmed in 28 countries (Yaninek and Herren 1988; Sileshi et al. 2019). CGM feeds only on cassava, primarily attacking young leaves, preventing their development and reducing photosynthetic capacity (Parsa et al. 2015) so that they remain small, pale and mottled. This pest has been successfully controlled in the past through the introduction of phytoseiid mites as a form of biocontrol (Robert et al. 2016). Figure 1 shows a map with cassava growing regions in the world with indication of the geographical extent of the major cassava pests.

## Modelling approaches for the surveillance, detection and control of cassava pests

We focus here on conceptual models of system dynamics rather than statistical methods. The amount of research on the surveillance and control of a given pest is often directly proportional to the economic importance of that pest. Most of the models developed for cassava have therefore focussed on CMD, CBSD, CM and CGM. Although CBB is economically important, not much attention has been given to it from a modelling perspective and little has been done on other, less economically important arthropod, virus and bacterial pests.

### Surveillance and detection

Over the past decades, the rate of introduction of non-native species of pests has increased substantially across the globe. This has affected the productivity and associated ecosystems of a great number of crops, including cassava (Graziosi et al. 2016; Parnell et al. 2017; Carvajal-Yepes et al. 2019). The introduction of invasive pests is generally attributed to an increase in international trade and the movement of people as well as climate change. The intensification of cropping systems and poor crop husbandry then exacerbate the situation (Montemayor et al. 2015; Graziosi et al. 2016; Delaquis et al. 2018). In the case of cassava, these invasive biotic threats have severely impacted yield (Graziosi et al. 2016) and in many regions this has resulted in a reduction the area of cassava grown (Otim-Nape et al. 2001). Invasive pests, therefore, can have severe impact on rural livelihoods, cassava-based industries, local economies and food security (Graziosi et al. 2016).

To tackle these new invasive pests effectively, it is widely acknowledged that biosecurity needs to be strengthened (Graziosi et al. 2016). Potential new environments and pathways need to be identified and risks mitigated. Pest risk maps are an important resource for developing appropriate risk mitigation measures such as phytosanitary regulations, the establishment of pest-surveillance networks, and the development of emergency response plans (Parsa et al. 2015). Correlative models built from species occurrence data, climate variables and host distribution provide an effective means to develop these maps. Montemayor et al. (2015) demonstrated a correlative modelling approach to predict the potential for invasion and spread of cassava lace-bug (*Vatiga spp.*). Similarly, Parsa et al. (2012) developed a dispersal risk for the cassava mealybug using a CLIMEX distribution model. This work predicted that dispersal risk was limited by cold stress and high rainfall in the wet tropics. More recently, Yonow et al. (2017) advanced this model by considering additional variables such as irrigations and host distribution. This resulted in a more accurate prediction of areas at risk of dispersal from CM in Asia, South America, and Africa. Later, Parsa et al., (2015) also predicted the potential distribution of cassava green mites (*Mononychellus tanajoa* and *M. mcgregori*) using a maximum entropy approach. These methods are useful for highlighting regions at risk to certain pests, but they need to be interpreted carefully as they do not explicitly account for the underlying biotic interactions (Montemayor et al. 2015). Geographic distributions are more likely to be accurately predicted if the model variables are more purposely selected based on the ecology and biology of the species. This was demonstrated by (Campo et al. 2011) who used ecological niche modelling to predict the potential geographic distribution of four threats to cassava, (whitefly, green mite, cassava mosaic disease and cassava brown streak disease) using known locations of each pest to characterize the environmental profile and potential distribution of each threat.

Improved use of quarantine and border inspections can reduce the risk of entry to new regions (Martin et al. 2016; Parnell et al. 2017); however, pre-emptive measures such as these do not avert all epidemics. Effective surveillance schemes within the agricultural landscape, are therefore essential. For emerging pests, surveillance is generally conducted to (i) determine whether a threat is present (detection), (ii) gather information to understand the nature and extent of the problem (estimation), and (iii) to identify as many infected sites as possible to implement control (targeting).

International guidelines emphasize the importance of statistical methods to inform surveillance (FAO 2006). Parnell et al. (2015) describe some generally applicable statistical methods for determining the incidence that an epidemic has truly reached when it is first detected. These methods account for the rate of epidemic increase as well as the intensity and frequency of sampling (Parnell et al. 2012, 2015; Bourhis et al. 2019). For detection, it is also important to know where to sample. Geostatistical methods have been proposed to address this (Lecoustre et al. 1989; Tubajika et al. 2004; Stonard et al. 2010). For example, (Bouwmeester et al. 2012) used regression kriging to interpolate the point-based surveys in Rwanda and Burundi and predict the spatial distributions of different measures of Cassava mosaic disease. They used environmental and sociological variables as fixed effects (or predictors) in their model and found that the environmental variables that were significant accorded with those that affected the location of the host crop and the abundance of the white-fly vector. Although these approaches can account for host variability, they are static in nature and so do not fully account for the landscape connectivity or the epidemiology of the threat.

Risk based sampling approaches, based on host distribution and the dispersal characteristics of the pest have been explored successfully in other systems (Hyatt-Twynam et al. 2017). In the case of threats to cassava, sampling efforts have been designed to gain insight into factors driving the spread and abundance of the pest and so have not focused on risk-based detection. For example, sampling has been undertaken to understand the impact of variety and crop area (Otim-Nape et al. 2001; Emily et al. 2016), environment (Legg and Ogwal 1998; Wudil et al. 2017), vector (Legg and Ogwal 1998; Mwatuni et al. 2015; Eni et al. 2018) and anthropogenic factors, such as trade and movement of contaminated cuttings, driving spread (Legg and Ogwal 1998; Mwatuni et al. 2015; Graziosi et al. 2016; Minato et al. 2019), set up a sample design to determine a baseline for the incidence of Sri Lankan cassava mosaic virus in Cambodia and Vietnam following its first detection in the previous year in Eastern Cambodia (2015). This type of surveillance effort is extremely important to determine severity, identify pathways for spread and provide recommendations for control. The design of where to sample, was somewhat risk based, in that it focused on districts with high density of production, however it does not take account of any other epidemiological factors.

In practice, many surveillance programmes ignore the processes that determine the dynamics of the pest spread (Parnell et al. 2017). To address this, several researchers have proposed using stochastic spatially explicit models to determine where it is best to sample (Gilligan and van den Bosch 2008; Parnell et al. 2010, 2017; Cunniffe et al. 2015b; Thompson et al. 2016). These models can be used to simulate realistic patterns of epidemic spread through heterogeneous landscapes, allowing for environmental conditions, uncertainties in the current levels of knowledge about the epidemic (e.g. transmission efficacy and dispersal characteristics) and human-mediated pathways for spread. Human-mediated spread is of particular relevance for cassava pests, such as cassava mosaic virus and cassava brown streak disease, where seed exchange mechanisms have facilitated their rapid spread across countries in Asia and Africa (Legg 1999; Legg et al. 2011, 2015; Mwatuni et al. 2015; Delaquis 2018). Analysis of seed networks as potential epidemic pathways can help to identify key locations for sampling and mitigation of pathogens in seed networks, and to evaluate the roles of different actors (Delaquis 2018).

There are practical constraints to surveillance that must also be addressed. A shortage of suitably trained personal and logistical difficulties of accessing sites affect the number of assessments that can be made and their locations (Quinn 2013; Carvajal-Yepes et al. 2019) In many cases, sampling is restricted to crop areas that are easily accessed from main roads (Mutembesa et al. 2018). Another issue is the ability to diagnose a pest problem. Infection can be difficult to diagnose both because of lack of training and also the cryptic nature of many pests (Awoyelu and Adebisi 2015; Minato et al. 2019). For example, (Minato et al. 2019) used PCR-based diagnostics to detect Sri Lankan cassava mosaic virus in Cambodia and found that 14% of infected plants did not express symptoms.

PCR-based diagnostics haven proven accurate for detecting cassava viruses (Abarshi et al. 2010; Minato et al. 2019), but this can be costly for large field surveys (Abarshi et al. 2010). Accurate and timely diagnosis of visible symptoms by non-experts offers great promise for improving the early detection of threats to cassava (Mutembesa et al. 2018). Model-based tools, deployed for example on smart phones, have been proposed to aid non-experts in diagnosis. These have used fuzzy expert systems (Awoyelu and Adebisi 2015) and multi-criteria decision making (Goodridge et al. 2017). Similarly, image-based detection methods have been proposed and proliferated during the last years using approaches such as machine learning, deep learning (Barbedo 2017; Ramcharan et al. 2017, 2018; Ferentinos 2018; Segun et al. 2019; Arnal Barbedo 2019; Tusubira et al. 2020), and image processing (Powbunthorn et al. 2012; Majumdar et al. 2014; Ninsiima et al. 2018). Automating the process of diagnosis is argued to give more accurate and standardised results (Quinn 2013). However the true strength of surveillance measures that integrate model-based prediction, expert assessment and citizen science will only be realised if backed up by regional diagnostic hubs, data management, risk assessment, and communication protocols as advocated by Carvajal-Yepes et al. (2019). Modelling has helped uncovering methods for stronger biosecurity measures, better surveillance and strategies for early pest detection; however, it still needs to be strengthened with the active collaboration of farmers, managers and policy makers to increase its effectiveness and timeliness.

### Cassava pests’ control

How to deploy control for cassava pests has been a subject of study for many decades (Herren 1994; Thresh et al. 1998; Jeger et al. 2006; Legg et al. 2006, 2015, 2017; Rapisarda and Cocuzza 2017). Control strategies include host-plant resistance, chemical and biological control, integrated pest-management, phytosanitation, intercropping, cultural practices and clean seed systems. Using more than a single strategy to manage or control pests often brings better control than using a single strategy (Tonnang et al. 2017). Cassava is not the exception and strategies trying to understand how to better optimise control through the inclusion of several methods have been developed for many years (Thresh 2004; Jones 2004; Jeger et al. 2004; Nutter 2007; Sastry and Zitter 2014; Rapisarda and Cocuzza 2017).

In this section we examine modelling work developed to inform and optimise control and management of cassava pests. Work dedicated to control virus diseases has historically focused on breeding for resistance, phytosanitation, the use of chemicals and clean seed systems, so we start this section looking at models developed in these areas. We then focus in studies analysing arthropod pests which, have primarily focused on biocontrol. In general, little has been done around control strategies for human-mediated dispersal, which is key in the long-distance spread of pests. Therefore, we analyse studies looking at this form of dispersal at the end this section.

#### Resistance

Breeding hosts for vector or pathogen resistance has been recognised as a key strategy for control of virus diseases (van den Bosch et al. 2007). Efforts to breed resistant cassava varieties have been developed for several decades now (Ceballos et al. 2012); however, modelling efforts that can reproduce the evolution of resistance in plants and pathogens is one of the key challenges in the modelling of plant diseases (Cunniffe et al. 2015a). Here we present studies focused on the use of cassava resistant and tolerant varieties as a form of control. In cassava resistant varieties, the host has the ability to limit pathogen multiplication while tolerant varieties only reduce the negative effects of infection (Pagan and Garcia-Arenal 2020).

Sometimes, when a pathogen spreads in resistant cultivars, the pathogen spread within and among cuttings stays relatively low and does not become fully systemic, thus some of the cuttings propagated from infected plants may revert to healthy plants (Fargette and Vié 1995). This phenomenon is known as reversion. Fargette and Vié (1995) investigated the effects of resistance, cutting selection and reversion on epidemic severity over time. They found that when either reversion or cutting selection occurred over several consecutive years, although the severity increased during the first few cycles, the disease reached an equilibrium with limited yield losses, concluding that the use of these two strategies simultaneously may help controlling the spread of ACMD.

Another study went beyond cutting selection and investigated the effects of different strategies on the control of virus cassava diseases (van den Bosch et al. 2007). This study shows that if resistance reduces infection transmission then, infection does not impose selection on the virus to evolve. However, if one breeds for tolerance, where plants retain a high virus titre but are symptomless, selection for strains with higher virus titre occur rendering resistance redundant. This would indicate that, although resistance is a very important form of control in the spread of virus diseases, care has to be taken when deploying resistant varieties that may be vulnerable to the evolution of virulent strains (Seal et al. 2006a; Nutter 2007). Also analysing resistant varieties, Magoyo et al. (2019) modified a model by Holt et al. (1997), including one CMD resistant and one susceptible cassava varieties. In this study both varieties became infected over time when no other control was undertaken, deeming the resistant varieties unsuccessful as a form of control in the long term.

In terms of molecular mechanisms of plant defence against viruses, (Neofytou et al. 2016) investigated the interactions between two viral strains and a single host. They investigated how RNA interference (the ability of host cells to recognise and degrade the messenger RNA of invading RNA, for example) may influence or explain cross-protection (the process by which infection of the plant with one virus can prevent or interfere with the subsequent infection by a second virus of the same family). Their results show that when two viruses “antagonise” each other, for sufficiently high “warning rates” provided by the plant immune system through RNA interference, not only can one minimise the spread of a specific virus, but the overall infection can be reduced. Conversely, if the two viruses are immunologically unrelated and co-infecting the same plant, they can indirectly promote each other. This can happen by, for example, making the cells that the first virus cannot infect anymore, more susceptible to the second infecting virus.

The mechanisms that confer tolerance and resistance to cassava plants are often complex. In this aspect, modelling has contributed a great deal in the understanding of how host resistance and tolerance mechanisms can provide long-term pest control. Modelling also shows that using resistant and tolerant varieties as a single pest control strategy can have varied and sometimes contradicting results. Therefore, one should be careful in the deployment of these varieties to ensure the effectiveness of control. Some ways to enhance control while deploying resistant varieties can include insecticide spraying, the use of clean cuttings and phytosanitation in conjunction with resistant varieties (Magoyo et al. 2019). More research is needed in the modelling of molecular mechanisms of plant defence similar to the model developed by (Neofytou et al. 2016) to better understand how resistance can be used for the management of cassava pests.

#### Phytosanitation and chemical control

Phytosanitation can be defined as the activity of improving the health status of cassava cuttings and decreasing the availability of sources of infection by the removal of diseased cassava (roguing) and the use of disease-free stem cuttings (Thresh et al. 1998).

In the early 1990s several models aiming to inform the control of ACMV spread were developed analysing the efficacy of methods such as roguing, planting of clean cuttings and reversion (Fargette et al. 1994). One of these simulation models showed that when reversion does not occur, and cuttings are not selected preferentially from healthy plants, disease incidence increased over time. Conversely, when either reversion, cutting selection or both strategies were adopted, the disease incidence could reach equilibrium values in cassava resistant varieties. Looking at a more general model of plant virus disease with roguing and replanting Chan and Jeger (1994) showed through a SEIR (Susceptible-Exposed-Infected-Removed) differential equation model with post-infectious plant populations that roguing as a form of control has no advantage when applied in the post-infectious phase. However, when applied at low contact rates and when the plants just become infectious roguing can result in disease eradication. This model also shows that at high replanting rates, the disease is more difficult to eradicate. In this model however, the vector population is not explicitly considered.

Once again we mention the model by (Holt et al. 1997) as besides describing the epidemic development of ACMV, it examined the efficacy of methods of control such as roguing and the use of clean cuttings. Their model shows that the use of clean cuttings is effective when infected cuttings are the main drivers of disease but roguing becomes important when the disease is vector driven. Moreover, when infected cuttings were planted in a frequency-dependent manner, roguing did not reduce disease incidence but it helped preventing the whole crop from becoming infected.

A model including the transmission mode of the vector was developed to understand what was the effect of roguing and vector management in disease control (Jeger et al. 1998). The model is a differential equation SEIR-type model for the host population and a SEIR-type model for the vector. This model shows that roguing is an effective mean of control only for non-persistently transmitted viruses, i.e. for viruses that are restricted to the stylet of the vector and can be transmitted for only a few minutes, and at a low vector-population density. This model also shows that the best way to prevent an epidemic is to decrease the vector-population density. Roguing is also ineffective when there is a continuous flow of viruliferous vectors and no epidemic threshold.

A set of compartmental differential equation models focusing on vegetatively propagated virus diseases and mosaic disease looked at the use of roguing (Chan and Jeger 1994), continuous cultural control (i.e. replanting and roguing) with a time delay due to disease latent period (Zhonghua and Yaohong 2014), discrete cultural control (Luo et al. 2015), pulse roguing with and without a periodic environment (Gao et al. 2016; Rakshit et al. 2019), and a mixture of insecticide/roguing control (Al Basir et al. 2017; Bokil et al. 2019).

Some of these models are more theoretically focused than others but all provide insight into the dynamics of the infected and susceptible host populations under different control scenarios. For example, the model with continuous cultural control (Zhonghua and Yaohong 2014) showed that the most influential factors on the basic reproduction number of the disease, R_0_ are the transmission rate and the replanting rate while the population dynamics is most influenced by the transmission, harvesting and the replanting rate. The model with periodic environment and pulse roguing (Gao et al. 2016) showed when the infection rate is high it may be impossible to eradicate the disease by simply roguing, that increasing the planting rate is bad for disease control and that when compared to impulsive control, where impulsive control refers to the implementation of periodic replanting of healthy plants or removing infected plants at a critical time, continuous control may overestimate infectious risk. Rakshits’ et al. (2019) model is focused on mosaic disease and its’ structure is similar to the other models mentioned here. However, in this case the model analyses how impulsive periodic roguing impacts the level of control obtained. This model shows that roguing is most useful and cost effective in controlling mosaic disease when applied at high roguing rate and short time intervals. However, as infection rate depends on vector densities, variable roguing and interval rates should be studied for maximum removal of mosaic disease in fields. During maximum disease incidence, roguing rate should be higher and time interval shorter but time interval should increase as eradication process takes place.

(Bokil et al. 2019) developed a model with two different replanting strategies to combat ACMV when control is administered through roguing and insecticide application. The two replanting strategies are (a) replanting stem cuttings from both, susceptible and infected plants, and (b) infected plants are replanted based on a fixed frequency of selection. The model showed that optimal control strategies for both replanting scenarios can be found in both cases, but they differ between each other and are not directly comparable. This model also shows that a strategy combining roguing and insecticide performs better than single control.

Insecticide alone is rarely used to control cassava pests in Africa both because it is expensive to use and the effect would be limited due to the lack of control in neighbouring plots and the development of insecticide resistance (Seal et al. 2006b). However, insecticide spraying as the main pest control resource has been studied in the production of *Jatropha curcas,* a close relative of cassava which is cultivated commercially as a biofuel source and is also affected by mosaic disease.

Venturino et al. (2016) developed a host-vector population model with a temperature-dependent vector population growth. The results of this model show that there is no benefit in applying insecticide during the first 10 days of the infection, but afterwards spraying should be applied for 3 months to achieve disease eradication. Insecticidal soaps have also been used in the control of mosaic disease on *J. curcas.* These soaps aim to block the spread of whitefly-borne infection by decrease the number of eggs being laid on a host and disabling adults from flying (Roy et al. 2015). Roy et al. (2015) used the significant similarities between mosaic infections of cassava and Jatropha plants to parameterise and develop a mosaic disease model to investigate the impact of continuous and pulse spraying strategies for the application of insecticidal soap to eliminate vector population concluding that impulsive spraying provides better control than continuous spraying and can lead to disease eradication.

Al Basir et al. (2018) modelled the spread of mosaic disease with the application of control through insecticides and nutrients as a function of the level of farmers’ population disease-awareness. Their model shows that an increase in population disease-awareness associates with a higher level of insecticide use which can then translate in possible disease eradication.

A model linking ACMV and the whitefly not only included parameters related to spraying and roguing, but also looked at transmission rates and level of host-resistance (Jeger et al. 2004). Their analysis indicates that roguing applied once per month in combination with a host showing a modest level of resistance can lead to disease eradication, while combining only roguing and insecticide applications is less effective.

In general, most of the modelling work done around the way insecticides and phytosanitation should be applied has been theoretical, perhaps, to understand what strategies are the most likely to work and achieve a good level of disease control (Bokil et al. 2019). These models provide general guidance on how to avoid high replanting rates by using roguing as a strategy for control while looking for varieties that may decrease transmission rates. The use of insecticides as a form of pest control in Africa is largely discouraged due to the high cost it represents to subsistence farmers, the potential negative consequences it may have in other forms of biocontrol and the development of insecticide resistance if not well managed (Seal et al. 2006b). Nonetheless, the models presented here show that the use of insecticide in commercial crops such as *J. curcas* may lead to disease eradication. These insights are valuable in the deployment of control options for cassava pests not only in terms of the disease epidemiology but also in terms of the control application constraints such as cultivation type (subsistence vs. commercial) geography, environmental and human factors. A clear example is the application of insecticides for cassava pests. Although discouraged in Africa, insecticide application for cassava pests’ control in South Asia may be a viable option as cassava is a commercial crop in this region.

#### Clean seed systems

Transportation and trade networks are important pathways for the spread of pests (Brasier 2008; Liebhold et al. 2012), at the same time, cassava seed systems can be used as tools for the spread of clean cuttings and thus decrease virus disease pressure in regions covered by clean-seed established networks.

The concept of clean seed systems for vegetatively propagated crops in the context of disease covers a wide range of aspects amenable to modelling, such as issues of degeneration, reversion, resistance, vector control, phytosanitation and network analysis (Dyer et al. 2011; McQuaid et al. 2016; Delaquis et al. 2018). Despite this, little has been done in the study of networks as an aid in the control of spreading cassava pests and modelling studies have been infrequent, although some models do exist for other crops such as sweet potato and potato (Bertschinger et al. 1995; Thomas-Sharma et al. 2017a; Andersen et al. 2019).

Models of cassava seed systems can in the main be separated into those that consider a single field of clean seed, and those that consider the broader landscape. Models of a single field (Fargette and Vié 1995; McQuaid et al. 2016; Thomas-Sharma et al. 2017b) explore the circumstances under which a field remains viable, and act as a tool for identifying the impact of different control strategies. These models do not necessarily need to consider spatial aspects of disease dispersal (Fargette and Vié 1995; Thomas-Sharma et al. 2017b), but stochasticity may still be important through issues such as weather (Thomas-Sharma et al. 2017b) which is relevant for certain cassava diseases. However, the success of seed systems has also been shown to be highly dependent on external disease pressure (McQuaid et al. 2016, 2017b, 2017a; Thomas-Sharma et al. 2017b), so the context in which seed systems are located is recognisably important.

Such models of more than one field tend to be intrinsically spatial, including networks of interactions between growers (McQuaid et al. 2017b, 2017a), see also (Delaquis et al. 2018; Andersen et al. 2019). As a result, stochasticity in the network or spatial structure highlights the importance of variability in the sourcing of cuttings. Here, modelling has shown that although re-use of supply from within a field, along with small-scale local exchanges, dominates in terms of seed and virus dispersal (Delaquis et al. 2018; Szyniszewska et al. 2019), the potential for larger-scale movement allows for rapid spread of virus across a landscape (McQuaid et al. 2017b, 2017a). Modelling of cassava viruses, transmitted both through a whitefly vector and infected cuttings, in this way requires the consideration not just of a network of interactions or a dispersal kernel, but of a spatially explicit network in combination with vector dispersal. This is a recent issue that has begun to be explored in other systems as well (Sumner et al. 2017).

As mentioned previously, models of seed systems are intrinsically linked to the shared effects of improved varieties and phytosanitation (Fargette and Vié 1995; McQuaid et al. 2016, 2017b, 2017a; Thomas-Sharma et al. 2017b) reflecting reality (Legg et al. 2017). Indeed, frequent and effective phytosanitation has repeatedly been shown to be required to maintain these systems (Fargette and Vié 1995; McQuaid et al. 2016, 2017b, 2017a; Thomas-Sharma et al. 2017b). As a result, models of seed systems have allowed for aspects of grower behaviour (Thomas-Sharma et al. 2017b; McQuaid et al. 2017b; Andersen et al. 2019). While this is clearly important to the success of seed systems (Legg et al. 2017; Szyniszewska et al. 2019), modelling of behaviour has rarely been considered outside the field of human disease (see Funk et al. (2010)) and presents much opportunity for improvement.

Finally, while most models of cassava seed systems focus on the effects of disease, one previous study has considered the intrinsic effect of seed systems on gene flow, from the perspective of a vegetatively propagated crop compared to a sexually-reproduced grain crop (Dyer et al. 2011). This work warns of the risks of rapid introduction of genetically modified cassava and the possible effect on eradication of deleterious transgenes, highlighting the risk that regulation of exchange of cuttings could reduce the adaptive potential of the plant and prove unsuccessful for disease control. The effect of seed systems on the genetic potential of cassava is an issue where there is therefore much scope for improved modelling.

In general, modelling studies focused on the use of local and regional cassava networks for the deployment of clean seed systems can be helpful in the management of cassava pests. Nonetheless, these models should be developed in collaboration with land managers, farmers, and stakeholders. Equally, other interactions such as vector dispersal and climatic conditions should be considered if management is to be effective. It is important when assessing and developing these management methods to understand that agricultural practices will greatly influence the success of establishing clean seed systems.

#### Spatial management

Intercropping can often help in the dispersal control of vectored diseases and this has been widely studied. In Africa, cassava is a subsistence crop, therefore, it is often planted together with or next to other crops. One of the first intercropping studies in cassava (Fargette and Fauquet 1988) found that spatiotemporal patterns of CMD spread in cassava intercropped with maize were complex and inconclusive showing that intercropping did not always reduce the incidence of ACMV. Moreover, this study found that CMD incidence was sometimes higher in maize/cassava combinations than in cassava only. Contrastingly, other studies (Gold 1994; Fondong et al. 2002) showed that intercropping cassava with cowpea or maize reduced whitefly populations up to 50% and CMD incidence was reduced by approximately 20% (Fondong et al. 2002). A more general model analysing different cropping patterns (Jabłońska-Sabuka et al. 2015) showed that the use of intensive cropping patterns and resistant cultivars triggers aggressive virus adaptability concluding that to reduce virus adaptability and spread more diverse and less concentrated spatio-temporal patterns are needed.

Windbreaks are another form of spatial control which have been used in the control of ACMD. Windbreaks are regions where a fence, wall, line, or growth of trees or other vegetation such as hedges, hedgerows, vegetative barriers, or wind barriers are built or planted preventing the wind coming through with its force (Ying 2018). This, in principle reduces the whitefly populations and therefore, disease incidence. Using advection–diffusion equations, (Lawrence and Wallace 2011) analysed the spatiotemporal spread of ACMD and simulated the use of windbreaks and resistant varieties for its’ control. They found that installing windbreaks along the upwind edges of the field could help reducing the entry of new whiteflies into the field, thus, reducing the disease incidence. They also found that reducing the host density can help reducing the disease incidence and some configurations where empty strips were introduced also helped.

Combining the deployment of resistant varieties and crop management practices can be another form of control. (Parry et al. 2020) developed a spatially explicit model to understand how crop management practices combined with crop breeding strategies to suppress whitefly numbers influenced the dynamics of the whitefly populations. Their study shows that considering the spatial cropping regime (e.g. how many seasons in a year cassava is planted) and how much cassava was present spatially could greatly affect the effectivity of deploying whitefly resistant varieties. For example, they found that sometimes, for the purpose of suppressing whitefly populations, the cropping regime undertaken can effective without the need of deploying cassava whitefly resistant varieties.

The modelling studies presented here have shown that intercropping has inconclusive results in terms of pest management efficacy. Windbreaks, however, may help reducing disease incidence by decreasing the vector populations. Other spatio-temporal pest management methods, such as cropping regimes can provide effective control when applied correctly. The models presented here show that there is enormous variability in effectiveness of control provided by spatial management, and often it may have to be evaluated in a case-by-case basis as spatial and temporal scales, economic resources, climatic conditions, among others, will present different challenges for the establishment of a good pest management regime.

#### Biocontrol

Biocontrol has been widely applied to CM in Asia and Africa and CGM in Africa. The release of natural and introduced enemies and parasitoids has greatly helped controlling the population numbers of pests but the way that biocontrol agents interact with the pest and the way deployment takes place, both spatially and temporally, are still relevant subjects of study (Sileshi et al. 2019; Wyckhuys et al. 2019b; Aekthong and Rattanakul 2019).

To combat the CM attacks a parasitoid, the *Epidinocarsis lopezi* (DeSantis) was introduced in Africa and later in Asia. To assess the efficiency of this parasitoid in the biological control of CM several models were developed during the late 1970s and early 1980s (Cudjoe 1990). Using several population dynamic models of biocontrol (Gutierrez et al. 1988a) developed a CM specific model of their population dynamics with age structure and mortality due to natural causes and due to predation by the parasitoid. The model shows that during the dry season, the most important factor for the control of CM populations is the parasitoid *E. lopezi* while rainfall is the main control parameter during the rainy season. They conclude that the use of predators and parasitoids for the control of CM is very important. Another exotic parasitoid (*Epidinocarsis lopezi* (DeSantis)) was later introduced into Africa to aid the control of the CM, but unlike *E. lopezi* this parasitoid was unsuccessful. Gutierrez et al. (1993) built a model to understand why, although these two parasitoid species are related, one was successful in the aid of the CM control while the other was not. Their model shows that the dynamics of host size over time favours *E. lopezi* over *E. diversicornis* and the ability of finding hosts is 5 times better for *E. lopezi* among other environmental and biological factors. The authors conclude that although these factors were important in the regulation of CM by *E. lopezi*, other factors might be crucial in other systems.

In order to reduce CM populations in Thailand, green lacewings were introduced as their larvae can destroy over 100 mealybugs in a week by sucking fluids from their soft bodies. (Jankaew et al. 2019). To study lacewings effect on CM population numbers Promrak et al. (2016) developed a predator–prey model where lacewings were released continuously and periodically finding that if enough CM enemies are introduced, good control is achieved, whether lacewings are released continuously or periodically. Building on this model (Promrak et al. 2017) included age structure for the prey (the CM) and built an integro-differential model. The authors found two stable states, one where the CM population goes extinct after overcoming a population threshold for the predator level, and a second one where the CM and lacewings co-exist. Then, to understand the effect of temperature on the population dynamics, Promrak and Rattanakul (2017) built a cellular automata model and to analyse the level of biological control efficacy at different temperatures. They found out that although the introduction of lacewings helped controlling CM populations, as the temperature increases, the survival and fecundity rates of lacewings decreased, requiring a larger number of released adult green lacewings to obtain CM effective control. Beyond the population dynamics the authors considered that in this situation the farmer would have to consider accepting potential yield loss due to the CM as using lacewings as a form of control could be too costly (Promrak and Rattanakul 2017)*.*

Considering a mathematical model of delayed differential equations, (Jankaew et al. 2019) simulated the population dynamics of CM and green lacewings showing that the time delay in the reproduction of green lacewing larvae played an important role in controlling the mealybugs population. Thus, if the time delay is correct, the reproduction rate of lacewings can control the population of CM to acceptable levels but if the delay is larger than a found critical value the CM population oscillates within a given range and can also exhibit a chaotic behaviour.

Considering biological and environmental factors that can contribute to the control of CGM spread into West Africa, Gutierrez et al. (1988b) developed a model using as a reference their CM model. For the case of the CGM they discovered that the most important factors contributing to the population control of the CGM were rainfall, drought stress and food availability, as the natural enemies in the region did not influence the number of CGM.

To assess the viability of introducing the fungus *Neozygitis* cf. *floridan.* into Africa from South America as a form of biocontrol, Oduor et al. (1997) developed a susceptible-infected-contagious compartmental model between the CGM and this fungus maintaining a constant fungal per-capita transmission rate. The authors showed that the fungal pathogen can reduce the population growth of CGM when other factors such as low temperature, low food quality and other environmental variables are right for fungal development. However, the use of *N.* cf. *floridan* alone cannot drive local mite populations to extinction.

Using time series analysis from data collected in Benin and a mechanistic predator–prey model a population model of the CGM and the introduced phytoseiid predator *Typhlodromalus aripo* were examined (Hanna et al. 2005). They show that *T. aripo* has been able to persist and reduce the population density of CGM in a cassava field in Benin over a period of 7 years, although the mean density of both, predator and prey have declined over time. Analysing the two populations fluctuations they concluded that these may be attributed to predator–prey dynamics instead of being a product of abiotic factors, but more studies are needed to support this claim.

A metapopulation tritrophic model looking at the dynamics of cassava, CM and its’ natural enemies, and CGM and its’ natural enemies was developed to understand the interaction between these three populations in a heterogeneous landscape (Gutierrez et al. 1999). The model shows that high host habitat finding capacity by *A. lopezi* (the main introduced parasitoid of CM) can result in good suppression of CM and that the ability to find new habitat areas depends on patch density and degree of spatial heterogeneity. It also shows that the exotic predator *T. aripo* can control CGM whereas another exotic predator *T. manihoti* does not.

To study the potential use of biocontrol measures to manage CMD in Africa, Okamoto and Amarasekare (2012) modified the model by Holt et al. (1997) using their parameters obtained for CMD. Their approach assumes a differential equation model of the dynamics of the host, the vector, the host infecting pathogen and a pathogen infecting the vector, showing that conditions in which the vector-infecting pathogen can be established if the conditions are right, exist. For example, this model shows that highly efficient predators, parasitoids and highly virulent pathogens of the vector with high transmission rates are effective as biocontrol agents. It also shows that biocontrol agents can successfully reduce long-term host disease even if vector densities are not reduced. Finally, inundating a host-vector system with a natural enemy of the vector has little or no effect in reducing disease incidence, but a vector competitor can greatly reduce disease incidence. This model provides scenarios and insights of how biological control can be deployed in order to reduce CMD incidence. Another model looking at the effect that biocontrol can have on virus spread (Jackson and Chen-Charpentier 2018) used a system of differential equations with delay that included a parasitoid population that could predate on the virus-spreading vector. This model shows that predators must be introduced at a certain rate to provide a good level of disease. Equally, the model shows that periods where less infection is visible may be due to the delay between infection and symptom development.

In general, biocontrol models studying the interaction between arthropods such as CM and CGM and their hosts have proven, not just theoretically but in practice the usefulness of models to inform successful pest management campaigns. Unfortunately, these models cannot be immediately generalised, and in terms of management of virus diseases, the modelling of deployment of biocontrol agents has proven to be much more challenging. This is because several factors influencing the host-pest population dynamics can interfere with the success of biocontrol strategies.

#### Human behaviour in pest control

Generally, modelling of pest control is based on the study and understanding of the epidemiology and spread of the pest, the landscape structure, and the abiotic factors. However, human behaviour is often neglected. Pest control is only successful if it is adopted by the farmer. This is factor that has started to become an important aspect to consider modelling strategies for the optimal control of pests (Milne et al. 2018). In cassava modelling there are few recent examples of these attempts.

A model by (McQuaid et al. 2017b) defines cassava growers in two categories: loyal growers (those who obtain cuttings from the same sources over successive seasons) and disloyal growers (those who obtain their cuttings from different sources). This grower behaviour can limit or enhance the spread of CBSD. The model shows for example that when growers have a small number of suppliers or when they use the same suppliers the disease incidence is lower. Another model from some of the same authors (McQuaid et al. 2017a) studied the effect that aspects of the disease epidemiology such as disease pressure, communication among farmers and subsidies contributed to the adoption of improved plant material and the improvement of disease control.

Technology adoption and use of improved varieties by growers accustomed to a certain variety and taste is studied by Gomez Chamorro (2017). In this study, using a machine learning algorithm that measures the information that farmers have access to, the degree of interaction between farmers and their geographical locations, the effect that improved cassava varieties adoption from some farmers have on their peers. The co-variates used to understand the probability of adoption include socio-economic characteristics at the farm and municipality co-variates. This study shows that the average village adoption has a strong effect on the individual farm adoption. Another important factor is the distance between adopters and non-adopters. As the distance between these farmers increases the probability of adoption decreases.

Continuing their work on farmers’ knowledge of control interventions, Al Basir and Ray (2020) developed a model to study the dynamics of CMD with farmers awareness based roguing and insecticide spraying. Using numerical simulations, they searched for a strategy of optimal spraying and roguing through media awareness communications for cost-effective control. They suggest that awareness campaigns through radio and TV can help eradicating the disease.

An interesting approach on how the control of pests can influence the behaviour of humans was a study human health population (Burra et al. 2021). In this study, the authors analysed how the cassava mealybug invasion in Sub-Saharan Africa in the 1970–1980’s caused yield reductions of up to 80% on farms and across regions. The study showed that there was an association between cassava yield reductions, a decrease in birth rates and an increase in death rates. Once the parasitic wasp *A. lopezi* was introduced as a form of biocontrol in 1981, the cassava yields were restored, incrementing food security, and helping to improve human health indices.

In conclusion, the effect that human behaviour and practices have on the management of cassava pests should never be underestimated as most of the time, the management or control strategy success will depend on the adoption by the farmer. Luckily, more models are being developed where human behaviours and practices are taken into account. However, this is a field where modelling has a huge opportunity to grow and deliver.

### Host-pest interactions and dynamics

The host-pest dynamics of a system can depend on several biotic and abiotic factors. Here we include some of the most important factors where modelling has contributed to the better understanding of the dynamics of cassava with its’ pests. Some of the most relevant cassava pests are virus diseases including CMD and CBSD which are vectored by the whitefly; therefore, we start this section with models related to the dynamics of vectored disease transmission. We continue with methods of pest dispersal including the involvement of vectors in disease spread followed by a section on dispersal through multiple paths that can include for example vectored and human mediated dispersal. We finalise this section with a summary on models focused on the dynamics of a system where co-infection of the host by more than one pest takes place.

#### Vectored disease transmission

In a host-vector-pathogen system disease transmission may happen in diverse ways. Here we focus on cassava virus diseases as, to our knowledge, no models for CBB exist. A number of models aiming to understand the impact that transmission dynamics have in disease epidemiology have been developed (Jeger et al. 1998, 2018; Grilli and Holt 2000; Zhang et al. 2000b, 2000a; Madden et al. 2000; Roosien et al. 2013; Gandon 2018; Donnelly et al. 2019; Al Basir et al. 2020) and can be explored to better understand vectored transmission dynamics. Here we include some that can help us to better understand the dynamics of vector-transmitted cassava diseases.

The most important cassava viruses are CMD and CBSD. CMD is persistently transmitted by whitefly, which retains the virus for up to 9 days while CBSD is transmitted semi-persistently with virus retention times of not more than 24 h (Legg et al. 2011). Transmission mode has important consequences for the epidemiological dynamics of the disease, and can guide practitioners in developing optimal control strategies (Lapidot et al. 2014). A detailed study of the effects of virus-transmission mechanisms on disease epidemics was developed (Madden et al. 2000). They addressed the implications that vectored disease transmission can have in the epidemiology and control of diseases, depending on the vector-virus interaction. The basic principles of this study and many subsequent host-vector models of disease transmission are based on compartmental models of differential equations known as SEIR-SI models (see Box 1).

**Box 1:**
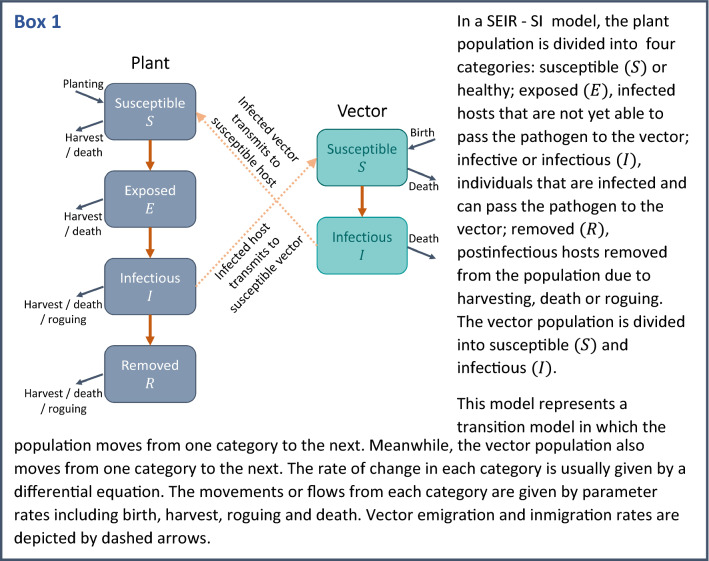
A schematic illustrating the components of SEIR-SI models

They used their results and used them to characterise CMD epidemics. As CMD is persistently transmitted, this model shows that to have a noticeable effect in epidemic control, a substantial reduction in the number of vectors per plant (e.g. through insecticides, cultural practices, etc.) is needed. The insights of this work can potentially be applied to CBSD and CBB and other vectored diseases. Based on the work by (Nault 1997) a summary explaining transmission characteristics associated with vectored plant viruses vectored transmission is found in Table 2.

**Table 2:** Transmission characteristics of plant viruses

Transmission characteristics	Non-persistently transmitted (stylet borne)	Semi-persistently transmitted (foregut borne)	Persistently transmitted, circulative	Persistently transmitted, propagative
Acquisition time	Seconds, minutes	Minutes, hours	Hours, days	Hours, days
Retention time	Minutes	Hours	Days, weeks	Weeks, months
Latent period	No	No	Hours, days	Weeks
Virus in vector haemolymph	No	No	Yes	Yes
Virus multiplies in vector	No	No	No	Yes
Transovarian transmission	No	No	No	Possible

Disease transmission is also affected by the vector’s feeding period. Using parameters for African Cassava Mosaic Virus (ACMV) transmission, Grilli and Holt (2000) developed a model for variable vector feeding time. They discovered that for inefficient virus transmitters variability in the vector feeding period can reduce or increase the epidemic development. This is relevant for both CMD and CBSD, where only a small percentage of vectors acquire the virus from infected plants even after long feeding periods (Grilli and Holt 2000; Maruthi et al. 2005). Disease transmission and prevalence can be also affected by the latent time of infection inside the vector after acquisition of the virus and the incubation period of the disease on a newly infected plant. Using a delay differential equation model and parameters of Cassava Mosaic Disease from (Holt et al. 1997; Jackson and Chen-Charpentier 2017; Rakshit et al. 2019), Al Basir et al. (2020) found that delays in the latent and incubation periods for the vector and the plant respectively have a big effect on the disease dynamics, concluding that biocontrol, genetic engineering, insecticides or any control measures that can delay the incubation delay period in plants can be used to drive the system to a disease-free equilibrium.

Vectored disease transmission can affect vector fitness or behaviour, which in turn, influences disease spread. For example, Holt et al. (1997) developed a model that predicted that if ACMV changed the fitness of the vector by increasing its’ population growth rate, then the pathogen spread rate was significantly affected. When virus infection led to increased fecundity; vector spatial aggregation was promoted. Considering that whiteflies prefer to feed on infected cassava plants, Zhang et al. (2000a) developed a model for the spread of CMD in Uganda. This model predicted that vector aggregation led to a reduction of within-crop disease incidence but might promote increased emigration rates of infected vectors to surrounding crops. This was in accordance with experimental results (Mauck et al. 2012). Another study including vector aggregation and whitefly dispersal behaviour in CMD (Hebert 2014; Allen and Hebert 2016) showed that these two factors can affect the rate of disease spread and the potential CMD outbreaks.

Knowing the epidemiological parameters in the field are time consuming and often hard to accurately measure as external variables are impossible to control. (Donnelly et al. 2020) used a method to estimate the CBSV vector retention period, acquisition period and inoculation period parameters for *B. tabaci*. To do this they matched laboratory experimental data with theoretical parameters using a vector dynamics population model and stochastic simulations. They found that whitefly retention time of CBSV is much shorter than previously assumed, offering a new perspective on the epidemiology of CBSD. This way of obtaining parameter estimates can be used to enhance the prediction of epidemic risk and strategies of control.

These models show that the way the host, the vector and the virus interact has an enormous impact, not only in the disease spread characteristics but also in the disease prevalence. Factors such as transmission mode, vector feeding periods, incubation periods, vector and host fitness parameters modified by the virus, have important consequences for the epidemiological dynamics of the disease and can guide policy makers in developing better management strategies. Additionally, new methods to obtain accurate epidemiological parameters, often hard to obtain experimentally, can be developed through modelling as exemplified by Donnelly et al. (2020).

#### Pests spread and dispersal

Modelling studies of cassava pests’ dispersal have primarily focussed on the whitefly *Bemisia tabaci*, the vector of CMD and CBSD, the spread of CMD itself, and the movement and distribution of the CM. More recent models have included human-mediated spread, but the focus has continued to remain on CMD, CBSD and to a lesser extent, CM. As far as we are aware, no models of dispersal have been developed for other pests, however, many generic models on disease dispersal can inform the population dynamics of specific pests, as long as they are correctly parametrised, and their dynamics captured by the model.

The earliest models of CMD dispersal were developed in the late 1980s after experimental studies on the incidence and spread of CMD in Africa were developed. Fargette et al. (1986) developed a model of CMD whitefly-vectored disease spread to develop control mechanisms, while Lecoustre et al. (1987, 1989) developed a geo-statistical model of the ACMV spread in Côte d’Ivoire. This work was later extended by Fargette et al. (1993) who analysed and fitted data of the temporal progress of ACMV in Côte d’Ivoire to understand which variables influenced the epidemic spread, concluding that whitefly numbers and fluctuations in the temperature and radiation were the most influencing variables. Continuing this work, Fargette and Vié (1994) developed a model of the temporal spread of ACMV into plantings using data from Côte d’Ivoire observing that disease was mainly driven by age-dependent host susceptibility and seasonal variation in temperature.

Despite a better understanding of CMD dispersal, models developed by the end of the 1990’s did not always explain field data accurately, so models including vector aggregation were developed (Zhang et al. 2000a) and have continued to be an important feature in understanding CMD spread (Hebert 2014; Allen and Hebert 2016). Szyniszewska et al. (2017) used a geospatial approach to improve understanding of the CMD pandemic front in North western Tanzania. In this model, the authors were able to define a pandemic front of CMD by determining disease incidence and whitefly abundance in the regions where the rate of change between high and low incidence and vector abundance was highest, concluding that these were the two most important variables for pathogen dispersal.

Other models of virus dispersal, although not exclusive to cassava virus diseases have also accounted for the vector preference for infected or non-infected hosts. For example Roosien et al. (2013) showed that vector change of preference for infected/uninfected hosts following acquisition of the pathogen can increase pathogen spread. Meanwhile Shaw et al. (2017) concluded that vector population growth rates are highly influential for virus spread rates, but the vector’s preference for settling on a host with a different infection status from itself, and the vector’s tendency to leave a host with the same infection status, led to increased pathogen spread. Sisterson and Stenger (2016), modelling variable birth and death rates affecting vector’s population size found that increasing vector mortality had a greater effect on pathogen spread than a model where the population size of the vector is fixed.

In terms of arthropod pests, a limited number of models on the spread of CM exist. However, (Gongora-Canul et al. 2018) considered the spatio-temporal dynamics of the mealybug *Paracoccus marginatus* Williams and Granara de Willink (Hemiptera: Pseudococcidae) on the cassava relative *Jatropha curcas* in the region of Yucatan, Mexico. The authors observed that at the beginning of the epidemic, a random dispersion pattern of mealybug-infested host existed. Over time, this pattern was overcome by anisotropic aggregation within host rows. This aggregation may then have aided mealybug dispersal across rows and over larger distances. Other examples previously mentioned looking at the dispersal risk of CM include the work of (Parsa et al. 2012) and (Yonow et al. 2017).

##### Multiple transmission pathways

As a corollary to the pest spread and dispersal section, we know that one of the primary dispersal pathways for cassava pests is through human-mediated movement of cuttings for planting. When cuttings are taken and used from an infected cassava plant, an infected plant will be established. For example, CMD, CBSD and CBB are horizontally vector-transmitted (the vector infects the plant by passing the virus/bacteria to the host while feeding) and vertically transmitted through infected cuttings. Meanwhile, for CM and CGM the movement of infested hosts can result in long-distance dispersal and the introduction of pests to areas previously pest-free.

Holt et al. (1997), modelled CMD transmission through vectors as well as through infected cuttings. They found that if all planted cuttings were virus-free, then, the only way in which disease could persist was through a high vector transmission rate or a large vector population. However, when they included a proportion of infected cuttings, three different scenarios emerged: disease elimination, healthy and infected plants, or ubiquitous infection. This seminal work led to the emergence of other models where the dynamics of the system included multiple disease transmission paths.

The efficiency of horizontal and vertical transmission depends on the virus strain in the plant. Strains that build up a high virus titre are easily picked up by the whitefly vector when feeding on the plant. Since high virus titres often goes paired with symptoms the grower will recognise these plants as infected and will not take cuttings to propagate the crop. Conversely, a plant infected with a strain that builds up a low virus titre is likely to be used as cuttings, but the vector will not easily pick up the virus while feeding. There is thus a trade-off between vertical and horizontal transmission.

Van Den Bosch et al. (2006) incorporated this trade-off and analysed which strains dominated the population under a range of disease control measures. They found that the removal of visually infected plants (roguing), selected for virus strains that build up a low virus titre; selecting tolerant varieties selected for virus strains with a higher virus titre. This is in agreement with a CBSD mixed-mode transmission study (McQuaid et al. 2017b) where it is shown that both, transmission via infected cuttings and human-mediated movement are highly important for disease dispersal.

Models of pest dispersal are important because they can help us establish likely scenarios of where the pest might establish and move towards. Integrating biological and epidemiological factors that account for the likelihood of dispersal such as vector preferences for specific types of hosts (e.g. infected vs. non-infected), vector population spatial configurations and favourable abiotic conditions have been crucial for the better understanding of pest spread. In the future models that can provide more holistic approaches integrating biotic and abiotic factors contributing to pest dispersal may be better suitable to explain dispersal patterns that are currently overlooked and therefore incorrectly predicted.

#### Co-infection dynamics

Host infection by more than one pest can result in synergy, co-existence, antagonism or cooperation among pathogens (Abdullah et al. 2017). To address this issue, Zhang et al. (2001), analysed a system where two mutually synergistic virus strains simultaneously infected cassava. This model was based on observations of African cassava mosaic virus (ACMV) and East African cassava mosaic virus (EACMV) in Cameroon (Fondong et al. 2000). The assumption was that by sharing the same host the two viruses would compete for the host’s resources limiting their ability to survive. They found that as virulence increased, the potential for co-existence decreased. Contrastingly, when virus transmission of dually infected hosts increased, the potential for co-existence increased. Although co-infection is known to occur frequently in cassava (Legg 2009; Vanderschuren et al. 2012; Zinga et al. 2013; Ogwok 2015), no other modelling studies of co-infection in cassava exist to our knowledge. The study of co-infection in plants remains as one of the most important challenges in the modelling of plant diseases (Cunniffe et al. 2015a).

Overtime, the acknowledgement of the role that the vector plays in the epidemiology of these virus diseases has been increasingly recognised. Virus transmission mode, feeding periods, spatial configurations and vectors preferences can have significant epidemiological and evolutionary consequences and these are being increasingly addressed in research modelling (Jeger 2020). However, links between the molecular and cellular that can explain the efficacy of disease transmission at larger scales are still needed to better understand. We expand on this in Sect. Future challenges and opportunities.

### Climate change impact on the management of cassava pests

How climate change will affect agricultural systems has become a frequently discussed and studied topic within the scientific community, however, its’ study in the context of agriculture and the management of agricultural pests is not new (Coakley et al. 1999; Garrett et al. 2011; Jones and Barbetti 2012). In terms of cassava there are some modelling examples as it has been highlighted that cassava can play an important role in climate change adaptation in Africa (Jarvis et al. 2012).

Global circulation models (Jarvis et al. 2012; El-Sharkawy 2014) were developed to analyse the impacts of climate change on staple foods. Results showed cassava will have remarkable resilience to climatic change, showing the ability to prosper with possible increases in average surface Earth’s temperatures of at least 1.5 ℃ or higher in the year 2030 and beyond. Equally, Gourdji et al. (2015) examined the vulnerability in the agricultural sector due to climate change in Latin America and the Caribbean. Using the EcoCrop niche-based model by (Ramirez-Villegas et al. 2013) they estimated among other crops, the cassava suitability to climate changes. They found that cassava in most regions from Mexico to the Andean region and the Southern Cone will maintain and increase its’ suitability due to the increasing temperatures.

Jarvis et al. (2012) also analysed the potential climate impact on whiteflies, CBSD and CM and how this then could impact cassava through ecological niche modelling. Their findings show that the geographical distribution of these pests will be impacted with new areas becoming suitable for them but also that some of the currently suitable areas may become less suitable. Their overall conclusion is that cassava will be resilient to future climatic changes providing the African continent with a good option for adaptation in a warmer world where most staple crops will face challenges. However, models looking at the potential whitefly distribution at different temperatures (Aregbesola et al. 2019; Aregbesola et al. 2020) point out that, even when climatic stress tends to negatively affect life history traits of whiteflies, these effects differ with the tolerance and potential climatic changes can modify the distribution and abundance of whiteflies as well as the environmental suitability for plant viruses. Moreover, Kriticos et al. (2020) analysed a time series data from East and Central Africa from 1981 to 2010 using CLIMEX, a process oriented climatic niche model, to assess the existing evidence linking climatic changes with *B. tabaci* abundance. They show that climatic conditions for the whitefly *B. tabaci* improved significantly in the areas where the pandemics had been reported providing some evidence that climatic changes attributed to the increase of whitefly abundance in East and Central Africa contributed to the increase of CMD and CBSD.

Additionally and despite the relevant findings on cassava suitability in a warmer world, another study analysed 13 climate change models based on the United Nations International Panel on Climate Change (IPCC) scenarios and looked at the suitability for the establishment of arthropod pests, thrips and whiteflies, showing that it will increase in many regions globally including South America, Southeast Africa, Madagascar, Coastal India and Southeast Asia (Bellotti et al. 2012). Although cassava is highly tolerant to draughts, a modelling analysis of cassava production data from Togo from 1978 to 2009 showed that, the most influential abiotic drivers of cassava yield in Togo were total rainfall, mean temperature and within-season rainfall variability (Boansi 2017). This study found that, beside other biotic variables, to increase future cassava yield in Togo, increasing the water supply during the main season and minimising water and heat stress during the lean season would be beneficial.

In conclusion, although it has been established that cassava production in a warmer and drier world is still possible, constraints in cassava production due to rainfall decrease and temperature changes as well as a potentially more favourable climate for the development of pests should be accounted for. Breeding varieties tolerant to draught, heat and common cassava pests and investment in low-cost irrigation systems, as well as a better integrative pest management system may help in making cassava a very suitable crop for a warmer and drier future.

## Future challenges and opportunities

Cassava has become a key staple and commercial crop in Africa, South America and South East Asia, but at the same time has been increasingly threatened by the incursion of invasive pests and the development of endemic diseases. Therefore, in this review, we took the opportunity to summarise and analyse the modelling developed around the surveillance, detection, management, and control of cassava pests identifying research gaps and opportunities as outlined below.

### Molecular advances opportunities for modelling

Developing resistant cassava varieties that can counter the attacks of one or more diseases (such as CMD and CBSD) effectively and over time is one of the main focuses to control diseases. However, little is known about all the dynamics between pathogens and with the host.

Surprisingly, few modelling studies make use of the rapidly increasing knowledge of the molecular mechanisms of plant defence against viruses even though they are common in medical epidemiology (Scherm et al. 2006). R gene-based defences and especially RNA silencing mechanism are becoming well understood at the molecular level (Calil and Fontes 2017). RNA silencing mechanisms are characterized by the ability of the plant to recognise and degrade the messenger RNA of invading RNA viruses or cause the methylation of target gene sequences and the genome of DNA viruses (Waterhouse et al. 1999; Calil and Fontes 2017). Models for this phenomenon on the molecular and cellular level have been developed. For example, Bergstrom et al. (2003) developed a basic model and showed how the silencing mechanism is a safeguarded against accidental damage due to activation of the mechanism by RNAs of the plant itself. Groenenboom and Hogeweg (2008) present a model that combines viral growth with RNA silencing. Viruses can overcome host antiviral silencing by encoding diverse viral suppressors of RNA silencing (Díaz-Pendón and Ding 2008). For the silencing suppression (Rodrigo et al. 2011) developed a model showing which type of suppression would evolve under what conditions.

Building such models at the molecular and cellular level into models describing plant level and even population level dynamics could for example help define ways for breeding for durable resistance or making durable and efficient use of cross-protection phenomena. In cross-protection a plant is inoculated with a mild virus strain to provide protection against a more aggressive virus stain. This is known to be an effective way of disease control. Neofytou et al. (2016) show that not only viral attributes but also the propagating component of RNA-interference and suppression in plants can play an important role in determining the level of protection. The modelled variables are however al at the level of the various types of infected plants. By adding the molecular level models, it should be possible to develop criteria about the molecular identity of viruses and that are good candidates for use in cross protection programs.

### Final remarks

The majority of cassava research has historically focused in the African continent, with records of mosaic diseases going as far back as the late 1800s (Storey and Nichols 1938). In South East Asia, cassava had been virtually pest free for most of its history until pests incursions occurred in the last 10–15 years (Graziosi et al. 2016). This has determined that modelling approaches follow a similar pattern with a large amount of work developed around the detection, control and understanding of CMD epidemics in Africa, and more recently also on CBSD. Nonetheless, as cassava has become a key commercial crop in South East Asia, a large amount of modelling has been recently devoted to the control of pests such as the CM and more recently CMD in Asia.

We summarised conceptual models of system dynamics for cassava pests considering surveillance and detection, methods of control, and host-pest interactions and dynamics. We then considered studies looking at the effect of climate change on cassava and its’ pests. Finally, we look at research opportunities that can take advantage of molecular advances to develop models that can link molecular and cellular knowledge into models describing plant and population level dynamics.

Research dedicated to the surveillance of cassava pests has primarily focused on developing sampling surveys to determine the incidence, severity and geographical extent of the pest, often ignoring the processes determining pest spread. General spatially explicit models that can help elucidate the underlying spread of pests do exist and these can help inform future sampling strategies for cassava pests.

Human-mediated dispersal is also a key component for pest spread in cassava, which has received some attention through the analysis of seed networks (Delaquis 2018) but requires more research and understanding. Practical constraints to surveillance also play an important role in the design of cassava sampling strategies and surveys as lack of trained personnel and difficulty of access to cassava locations affect the number of places and assessments that can be made (Quinn 2013; Carvajal-Yepes et al. 2019). Novel technologies including image-based detection and image processing are tools that can be integrated into model-based prediction, citizen science and expert assessment to provide better surveillance strategies and programmes.

Overall, great advances in the field of biosecurity, surveillance and detection modelling have been achieved in the last decades, making the detection of cassava pests more efficient. However, these advances are met with the challenge of an enormous increase of plant and produce movement between regions, countries, and continents. Additionally, socio-economic variables, climatic conditions, as well as local and regional customs are often ignored in models. Accounting for these variables is essential for surveillance strategies to be effective. These should be integrated into models to improve our chance of early control and eradication of cassava pests that may be introduced into new regions.

Methods of control of cassava disease have largely focused on CMD and to a lesser extent on CBSD. These methods have improved understanding of several phenomena such as disease spread, management and introduction of clean system networks, resistant varieties deployment and phytosanitation. Much of this work has been developed for the characteristics of the African continent where the diseases have been present for much longer and where a large proportion of the production system is a subsistence one. The cassava crop system in South East Asia differs greatly, as it is often exploited commercially with large areas of land planted as monocultures. This means that pests and disease dynamics will greatly differ between regions and this is something that modellers should consider when developing their models.

Modelling studies of CM and CGM have focused on biocontrol methods through the introduction of parasitoids where some of the most successful stories of cassava pests’ management can be found.

In terms of host-pest dynamics and their impact on cassava pest and disease spread, a number models have been developed over the years. Advances in the understanding of vectored disease transmission, vector behaviour and dynamics of pest spread have been made. However, more holistic approaches that look at the whole crop system are still needed to better understand the interactions and dynamics among host-vectors-diseases for viruses and hosts-pests for arthropods. These holistic models could include co-infections, genetic and molecular characteristics, climatic variables, socio-economic factors, and contrasting spatial configurations.

Modellers have taken little advantage of the fast-growing knowledge on molecular mechanisms of plant defence against pathogens. In particular, we see an opportunity for the better understanding of plant immune systems through the access of the rapidly increasing knowledge of molecular biology of plant and pathogens. This is a key area that modelling approaches should investigate as it will provide insights in the development of resistant cassava varieties and their spatio-temporal deployment.

Finally, a large amount of modelling work has been developed for other host systems, covering topics including surveillance, biocontrol, plant-virus epidemiology, co-infection dynamics and molecular biology in human epidemics. This knowledge should be taken advantage of to improve and advance the methods used for the control and detection of cassava pests. Additionally, areas looking at the interaction between multiple pathogens and cassava hosts due to biotic and abiotic constraints need further development for the better management of cassava pests.
